# Normal liver enzymes do not indicate safety from alcohol-related liver disease: evidence from a Korean nationwide cohort

**DOI:** 10.4178/epih.e2026004

**Published:** 2026-01-22

**Authors:** Yeon Woo Oh, Jun Young Park, Eun-Cheol Park

**Affiliations:** 1 Department of Biostatistics and Computing, Yonsei University Graduate School, Seoul, Korea; 2 Department of Preventive Medicine, Yonsei University College of Medicine, Seoul, Korea; 3 Institute of Health Services Research, Yonsei University, Seoul, Korea

**Keywords:** Alcohol drinking, Health behavior, Liver diseases, Alcohol-related disorders

## Abstract

**OBJECTIVES:**

This study examined whether individuals with consistently normal liver enzyme levels are protected from alcohol-related liver disease and investigated whether heavy drinking increases liver disease risk independent of normal biomarker status.

**METHODS:**

We conducted a nationwide cohort study using Korean National Health Insurance Service data (2002–2019), including 19,035 participants aged ≥40 years who maintained normal liver enzyme levels (aspartate aminotransferase ≤40 μ/L, alanine aminotransferase ≤40 μ/L, gamma-glutamyl transferase ≤63 μ/L for men and ≤35 μ/L for women) across multiple examinations conducted between 2002 and 2008. Participants were categorized as abstainers (≤1 time/mo), moderate drinkers (≤2 times/wk), and heavy drinkers (≥3 times/wk). Primary outcomes included incident liver disease identified using diagnostic codes. Cox proportional hazards models were used to estimate hazard ratios (HRs), with age-stratified analyses performed.

**RESULTS:**

Heavy drinkers demonstrated a significantly higher risk of liver disease than abstainers (HR, 1.73; 95% confidence interval [CI], 1.40 to 2.14), whereas moderate drinkers showed no significant association (HR, 1.06; 95% CI, 0.94 to 1.19). Consistent patterns emerged for middle-aged adults (40–69 years), with attenuated effects among participants aged ≥70 years. When alcoholic liver disease was analyzed specifically, both moderate (HR, 1.29; 95% CI, 1.05 to 1.58) and heavy drinkers (HR, 2.86; 95% CI, 2.09 to 3.91) exhibited significantly increased hazards.

**CONCLUSIONS:**

Despite normal liver enzyme levels, heavy alcohol consumption was associated with a significantly increased risk of liver disease. These findings challenge current reactive screening paradigms relying solely on biomarker abnormalities and support proactive alcohol counseling regardless of enzyme results. More sensitive strategies are needed for early detection of alcohol-related liver injury.

## GRAPHICAL ABSTRACT

[Fig f3-epih-48-e2026004]

## Key Message

Even among individuals who consistently maintain normal liver enzyme levels, heavy alcohol consumption significantly increases the risk of liver disease. These findings challenge the conventional assumption that normal biomarker results reflect hepatic safety. Proactive alcohol counseling should be provided to all heavy drinkers regardless of their liver enzyme status.

## INTRODUCTION

Alcohol-related liver disease is among the most devastating consequences of excessive alcohol consumption [1-5], accounting for 5.1% of the global disease burden and disproportionately affecting the productive population aged 15–44 years [6]. This issue is particularly concerning in Korea, where alcohol consumption remains deeply embedded in social and professional contexts [7]. The progression from early liver injury to clinically significant disease often occurs silently, with symptoms appearing only after substantial hepatic damage has already developed [8,9]. This delayed clinical presentation poses a critical challenge for timely intervention and effective prevention strategies [10,11].

Standard health screening protocols worldwide, including Korea’s national health examination system, typically rely on serum aspartate aminotransferase (AST), alanine aminotransferase (ALT), and gamma-glutamyl transferase (GGT) as primary indicators of liver function [12]. These biomarkers have long served as the cornerstone of liver health assessment in routine clinical practice [13]. However, emerging evidence suggests that these conventional markers have important limitations in accurately reflecting alcohol-induced liver damage, particularly in its early stages [14,15].

Reliance on liver enzyme testing fosters a fundamentally reactive approach to modifying drinking behaviors from both patient and clinician perspectives. Evidence indicates that heavy drinkers are more likely to alter their drinking patterns after receiving abnormal test results [16-18]. Similarly, clinicians tend to prioritize intervention and counseling for patients with elevated liver enzyme levels, identifying these individuals as the population at highest immediate risk [19]. However, the assumption that normal liver enzyme values reflect a healthy hepatic status remains uncertain. This clinical blind spot is further exacerbated by risk perception biases among heavy drinkers, who often overestimate their health status relative to objective indicators [20,21], thereby creating a dangerous disconnect between subjective health perception and ongoing disease progression.

We aimed to demonstrate that individuals with consistently normal liver enzyme levels are not necessarily protected from alcohol-related liver damage. By comparing liver disease incidence between heavy drinkers and abstainers within a Korean adult population maintaining normal liver enzymes, we sought to provide evidence that alcohol moderation should be recommended regardless of enzyme status. These findings are intended to inform more proactive preventive strategies that can be implemented before overt clinical manifestations emerge.

## MATERIALS AND METHODS

### Study population

This study utilized data from the National Health Insurance Service–National Sample Cohort (NHIS-NSC), a nationally representative cohort from Korea [22]. The cohort was initiated in 2002 with 1,025,340 participants (2.2%) sampled from 46,605,433 individuals included in the National Health Information Database, with annual additions of newborn infants. Sampling was conducted using a stratified random approach, with total annual medical expenses serving as the target variable. The dataset contains comprehensive information on participants’ insurance eligibility, medical treatments, healthcare institutions, and general health examinations. For the present study, we used version 2.2 of the NHIS-NSC. A detailed description of the cohort has been published elsewhere [22].

For this analysis, we applied sequential inclusion criteria to identify participants with sustained alcohol consumption patterns and initially normal liver function. First, we selected individuals who were aged 40 years or older in 2002 and who underwent at least 3 general health examinations between 2002 and 2008. Second, we restricted the cohort to participants who maintained normal liver enzyme levels (AST≤40 μ/L, ALT≤40 μ/L, and GGT≤63 μ/L for men and ≤35 μ/L for women) [23] across all health examinations. Third, we excluded participants with any diagnosis of liver diseases of interest before 2009. Based on Korean Standard Classification of Diseases (KCD) codes, exclusions included any history of alcoholic liver disease (ALD; K70), liver failure (K72), unspecified chronic hepatitis (B18.8, B18.9, B19, K73), liver cirrhosis (K740, K741, K742, K746), or liver cell carcinoma (C22), identified from claims data between 2002 and 2008. Fourth, to ensure an initially healthy study population, we included only participants with a Charlson comorbidity index score of 0 as of 2008 [24]. Finally, we restricted the analysis to individuals who reported consistent alcohol consumption patterns across all health examinations. After excluding participants with missing covariate data, the final analytic sample comprised 19,035 participants.

### Exposure (alcohol consumption pattern)

Participants’ alcohol consumption patterns were classified into 3 groups based on self-reported drinking frequency: abstainers, moderate drinkers, and heavy drinkers. Abstainers were defined as individuals who reported no alcohol consumption or drinking less than or equal to once per month. Moderate drinkers were those who reported consuming alcohol 2 or fewer times per week, whereas heavy drinkers were defined as those who reported drinking 3 or more times per week. To ensure consistency in exposure classification, only participants who reported the same drinking pattern across all health examinations conducted between 2002 and 2008 were included in their respective exposure groups.

### Outcome (liver disease)

The primary outcome was the incidence of liver disease, defined using KCD codes for ALD (K70), liver failure (K72), chronic hepatitis (K73), liver cirrhosis (K740, K741, K742, K746), and liver cell carcinoma (C22). Incident cases were identified using claims data from the index date of January 1, 2009, until the end of follow-up on December 31, 2019, or until death, whichever occurred first.

### Covariates

The association between alcohol consumption patterns and liver disease outcomes was examined after adjustment for potential confounding variables assessed at 2 time points. Demographic and socioeconomic variables, including gender, age (categorized as 40–49, 50–59, 60–69, and ≥70 years), and insurance percentile as a proxy for socioeconomic status (categorized as 0–30, 31–70, and 71–100%), were assessed as of January 2009. Health-related variables, including body mass index (BMI, dichotomized as <25 and ≥25 kg/m²), smoking status (never smoker, former smoker, and current smoker), physical exercise frequency (no exercise, 1–2, and ≥3 times/wk), and family history of liver disease (yes or no), were assessed at each participant’s most recent health examination conducted between 2002 and 2008.

### Statistical analysis

We conducted survival analyses to examine the incidence of liver disease from January 1, 2009, through December 31, 2019. Time to event was defined using an age-based time scale, with event time specified as the participant’s age in months at the first diagnosis of any liver disease of interest. Participants who died without a recorded liver disease diagnosis or who remained event-free through December 31, 2019, were right-censored at their age at death or their age at the end of follow-up, respectively. Deaths attributed to liver disease without prior diagnostic records in the database were censored, as these cases likely reflect diagnoses made outside the observation system rather than true incident events captured during follow-up.

Because participants were required to survive until January 2009 to be eligible for inclusion, the data exhibited a left-truncated survival structure. Accordingly, observed survival times represented conditional survival given survival until study entry, rather than marginal survival from birth. We addressed this left truncation by appropriately adjusting the risk sets in all survival analyses, with left-truncation times defined as participants’ ages at study entry [25].

Our analytical strategy proceeded in several steps. First, Kaplan–Meier survival curves were constructed according to alcohol consumption patterns, and log-rank tests were performed to evaluate differences in liver disease-free survival across groups. Second, Cox proportional hazards models were fitted to estimate hazard ratios (HRs) for the association between alcohol consumption patterns and liver disease incidence, with adjustment for potential confounders, including gender, insurance percentile, BMI, smoking status, physical exercise, and family history of liver disease. The proportional hazards assumption was assessed using Schoenfeld residuals. After identifying a global violation of the proportional hazards assumption, we conducted age-stratified analyses (40–49, 50–59, 60–69, and ≥70 years), within which no significant violations were detected.

Third, given that smoking is a well-established risk factor for multiple liver diseases [26], we performed subgroup analyses stratified by smoking status (never, former, and current smokers). In addition, liver disease outcomes were further categorized into ALD (K70) and non-ALD (K72, K73, K740, K741, K742, K746, C22), and separate analyses were conducted for each outcome category. As a sensitivity analysis, we performed an additional Cox proportional hazards analysis in which death from any cause was treated as an event rather than as a censoring mechanism. This analysis was intended to capture the potential impact of alcohol consumption on overall mortality, including deaths that may have resulted from undiagnosed liver disease or other alcohol-related conditions.

The statistical significance level was set at 0.05 for all analyses. All statistical analyses were performed using SAS version 9.4 (SAS Institute Inc., Cary, NC, USA) and R version 4.3.0 (R Foundation for Statistical Computing, Vienna, Austria).

### Ethics statement

This study was conducted in accordance with the ethical principles of the Declaration of Helsinki. All study protocols were reviewed and approved by the Institutional Review Board of Yonsei University Health System, Severance Hospital (IRB No. 2025-1733-001).

## RESULTS

The baseline characteristics of the study population are summarized in Table 1. Abstainers comprised 76.1% of the total population, whereas heavy drinkers accounted for only 2.6%. Notable differences in demographic characteristics were observed across drinking patterns. Among heavy drinkers, the proportion of men was substantially higher than that of women (96.3 vs. 3.7%). Smoking status showed a strong correlation with drinking patterns: 47.3% of heavy drinkers were current smokers, compared with only 6.3% of abstainers. Baseline liver enzyme levels increased with drinking frequency, with heavy drinkers exhibiting the highest mean levels of AST (24.1±5.6 μ/L), ALT (20.0±6.6 μ/L), and GGT (33.3±12.7 μ/L), followed by moderate drinkers and abstainers.

**Figure 1.**
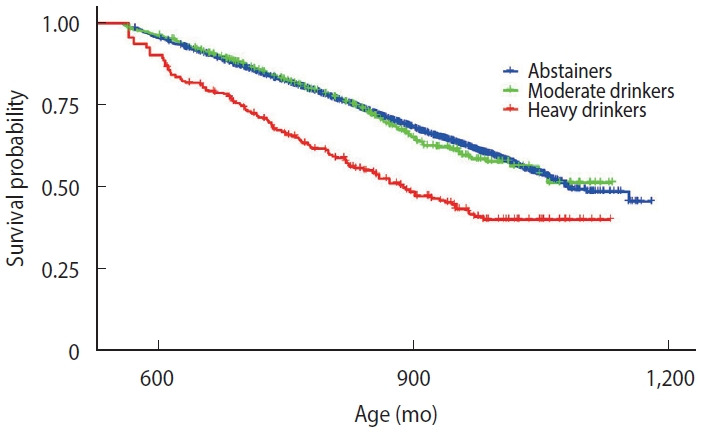
Kaplan-Meier curves for the incidence of liver disease by alcohol consumption frequency.

During the follow-up period, a total of 2,632 incident liver disease cases were identified, including 1,988 cases among 14,479 abstainers, 540 cases among 4,070 moderate drinkers, and 104 cases among 486 heavy drinkers. Figure 1 presents the Kaplan–Meier survival curves stratified by alcohol consumption pattern. The x-axis represents age in months and is truncated at 560 months (approximately 47 years) for illustrative purposes. Heavy drinkers (red line) demonstrated a markedly lower probability of liver disease–free survival compared with the other 2 groups. In contrast, the survival curves for abstainers and moderate drinkers were nearly indistinguishable throughout the follow-up period. The log-rank test indicated significant differences across the 3 groups (p<0.001). Although the Schoenfeld test suggested non-proportional hazards for drinking pattern (p=0.036; Supplementary Material 1), visual inspection of the Kaplan–Meier curves revealed approximately parallel survival trajectories, suggesting that this violation was unlikely to meaningfully affect the HR estimates.

Table 2 displays the results of the Cox proportional hazards models. Compared with abstainers, heavy drinkers demonstrated a significantly elevated HR of 1.73 (95% confidence interval [CI], 1.40 to 2.14), whereas moderate drinkers showed a non-significant increase in hazard (HR, 1.06; 95% CI, 0.94 to 1.19). Among the covariates, obesity (HR, 1.17; 95% CI, 1.08 to 1.28) and a family history of liver disease (HR, 1.27; 95% CI, 1.04 to 1.56) were also associated with significantly increased hazards.

Because a violation of the global proportional hazards assumption was detected by the Schoenfeld test (Supplementary Material 1), we present age-stratified results in Table 3. The proportional hazards assumption was satisfied within each age stratum (all p≥0.05). Consistent patterns were observed across the 40–49, 50–59, and 60–69 age subgroups, with heavy drinkers showing significantly increased hazards, whereas moderate drinkers did not differ significantly from abstainers. In contrast, among participants aged 70 years or older, neither moderate nor heavy drinkers exhibited a significant increase in hazard. Subgroup analyses stratified by smoking status demonstrated significant hazard increases among never smokers (HR, 1.50; 95% CI, 1.12 to 2.01) and current smokers (HR, 1.56; 95% CI, 1.18 to 2.05), while former smokers showed a moderate but non-significant increase in hazard (HR, 1.37; 95% CI, 0.90 to 2.09).

**Figure 2.**
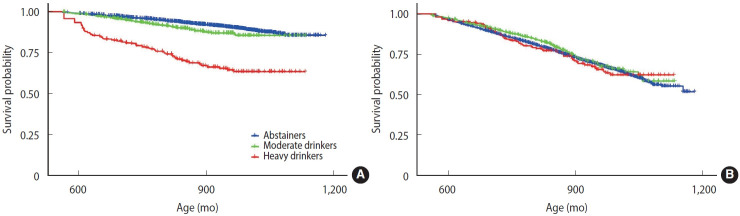
Kaplan-Meier curves for the incidence of (A) alcoholic liver disease and (B) non-alcoholic liver disease by alcohol consumption frequency.

When liver disease outcomes were further classified into ALD and non-ALD based on KCD codes, distinct patterns emerged (Table 4). For ALD, both moderate drinkers (HR, 1.29; 95% CI, 1.05 to 1.58) and heavy drinkers (HR, 2.86; 95% CI, 2.09 to 3.91) exhibited significantly elevated hazards compared with abstainers (Figure 2A). In contrast, for non-ALD outcomes, neither moderate drinkers (HR, 0.95; 95% CI, 0.83 to 1.09) nor heavy drinkers (HR, 1.20; 95% CI, 0.91 to 1.58) showed a statistically significant increase in hazard (Figure 2B).

In sensitivity analyses treating death as an outcome rather than a censoring event, similar patterns were observed (Supplementary Materials 2 and 3). Heavy drinkers demonstrated a significantly increased hazard (HR, 1.51; 95% CI, 1.27 to 1.80) compared with abstainers, whereas moderate drinkers did not show a significant association (HR, 1.00; 95% CI, 0.90 to 1.11). Although the global proportional hazards assumption was not satisfied in this model (p<0.001), age-stratified analyses confirmed that the proportional hazards assumption was met within all age subgroups. Consistent results were observed for participants aged 40–49 years, 50–59 years, and 60–69 years, whereas no significant increases were detected among participants aged 70 years or older for either moderate or heavy drinkers.

## DISCUSSION

In this nationwide cohort study of Korean adults, we observed that although all participants maintained consistently normal liver enzyme levels, heavy drinkers had a significantly increased risk of developing liver disease compared with abstainers. After adjustment for potential confounding variables, heavy drinking remained a significant independent risk factor for liver disease, whereas moderate drinking showed no significant association. These patterns were consistent across both current smokers and never smokers, as well as across most age subgroups, although the effect appeared attenuated among the oldest participants. When ALD and non-ALD were evaluated separately, the hazard of ALD was increased even among moderate drinkers, whereas the hazard of non-ALD remained non-significant even among heavy drinkers. The findings were also consistent when death was treated as an outcome rather than as a censoring mechanism.

Our findings are consistent with previous studies [27-29] indicating that liver damage can progress subclinically before abnormalities in liver enzyme levels become detectable. Previous clinical investigations have demonstrated fibrotic changes in liver tissue despite normal enzyme values [28], and advanced imaging techniques have identified increases in hepatic stiffness that remain undetected by routine blood tests [29]. Building on this literature, our study provides large-scale epidemiological evidence that individuals who were heavy drinkers at baseline, despite having normal liver enzymes at that time, face a substantially elevated hazard of developing liver disease.

Our subgroup analyses further highlighted heterogeneity in alcohol-related liver disease risk. Age-stratified analyses demonstrated consistent hazard increases among heavy drinkers across middle-aged groups. In contrast, no significant association was observed among adults aged 70 years or older, suggesting the possibility of survivor bias in older populations [30,31]. Individuals most susceptible to alcohol-related liver damage may have already developed disease or died before reaching advanced age, leaving a subgroup with relative resistance to hepatotoxic effects. Alternatively, older individuals may die from competing causes before liver disease develops, resulting in censoring that obscures potential differences between drinking groups. Notably, when ALD was analyzed as a distinct outcome, even moderate drinkers exhibited a significantly increased hazard. This finding challenges the widely held belief that moderate drinking is inherently safe and suggests that there may be no truly safe threshold for alcohol consumption, even among individuals with consistently normal liver enzyme levels.

Sensitivity analyses treating death as an outcome rather than a censoring event yielded similar patterns, further reinforcing the robustness of our findings. These results are consistent with prior large-scale studies demonstrating that alcohol-related harm extends beyond liver-specific outcomes to include overall mortality, particularly among individuals with sustained heavy drinking patterns [2,32].

Our findings can be applied to clinical practice within the framework of the health belief model (HBM) [33,34], which provides a conceptual basis for understanding health behaviors related to alcohol consumption [35]. According to the HBM, individuals are more likely to modify risky drinking behaviors when they perceive both a high susceptibility to alcohol-related harm and a high severity of its consequences [36]. However, normal liver enzyme results obtained during routine health screenings may reduce perceived susceptibility to alcohol-related liver disease and attenuate perceived severity. This misalignment between biomarker feedback and actual risk can hinder the internalization of health threats and weaken cues to action, thereby reducing the likelihood of adopting preventive behaviors such as alcohol reduction [37,38].

The clinical implications of our findings are substantial. Under traditional alcohol screening approaches [37,39], counseling interventions are often offered only after abnormal liver enzyme results are detected, resulting in a largely reactive rather than preventive paradigm. Instead of waiting for abnormal test results to serve as a cue to action [40], clinicians should proactively provide cues through personalized counseling on alcohol-related risks, thereby correcting the misperception that normal biomarkers imply safety. Evidence indicates that even brief counseling interventions delivered by physicians or nurse practitioners in routine primary care settings can effectively reduce alcohol consumption among high-risk drinkers [37,38].

This study has several strengths that enhance the validity and applicability of its findings. The use of a nationwide cohort and appropriate adjustment of risk sets for left truncation based on participants’ ages allowed for valid estimation of HRs across drinking patterns. In addition, the longitudinal design with extended follow-up enabled the capture of clinically meaningful outcomes beyond laboratory abnormalities. Importantly, by focusing on individuals with initially normal liver enzyme levels, this study directly addresses a critical blind spot in current clinical practice.

Several limitations should also be considered. First, the requirement that participants undergo at least 3 consecutive health examinations may have introduced selection bias. However, this criterion allowed for repeated verification of drinking patterns and liver enzyme levels over the 7-year baseline period, thereby strengthening exposure classification. Second, alcohol consumption was assessed using drinking frequency rather than quantitative intake because of limitations in the available health examination data. Although this precluded precise dose–response assessment, prior studies using the same Korean cohort data support frequency-based categorization, particularly the threshold of drinking 3 or more times per week for identifying individuals at high risk of adverse health outcomes [41]. Third, changes in drinking patterns during follow-up could not be assessed and may have influenced outcomes independently of baseline consumption. Nevertheless, inclusion was restricted to participants who consistently reported the same drinking pattern across multiple baseline examinations, suggesting relative stability over time. Fourth, we were unable to stratify participants by polymorphisms in genes such as *ADH1B* and *ALDH2*, which modify alcohol metabolism and susceptibility to liver disease [42,43]. However, because these polymorphisms influence alcohol-related outcomes partly through drinking behavior and tolerance [44], our phenotypic classification of drinking patterns may capture some underlying genetic variability. Fifth, we could not exclude individuals with previously diagnosed chronic hepatitis B or hepatitis C (B18.0, B18.1, B18.2) because of masking of corresponding KCD codes. Although such conditions may confound associations, their prevalence among Korean adults in 2009 was relatively low, generally not exceeding 4% for hepatitis B and 1% for hepatitis C [45-47], suggesting that any resulting bias is likely modest. Finally, generalizability may be limited outside Korea, where cultural norms surrounding alcohol consumption and genetic determinants of alcohol metabolism differ.

In conclusion, this study demonstrates that even among individuals who maintained normal liver enzyme levels during the baseline period, heavy alcohol consumption poses a significant health risk. These findings challenge the prevailing reactive paradigm that prioritizes intervention only after biomarker abnormalities emerge. By recognizing the limitations of liver enzymes as screening tools and addressing the gap between perceived and actual risk, clinicians may prevent individuals with normal biomarkers from underestimating the health consequences of alcohol consumption. Future research should focus on developing and validating more sensitive early indicators of alcohol-related liver damage and on evaluating targeted interventions that address risk perception among heavy drinkers with normal biomarker profiles.

## Figures and Tables

**Figure f3-epih-48-e2026004:**
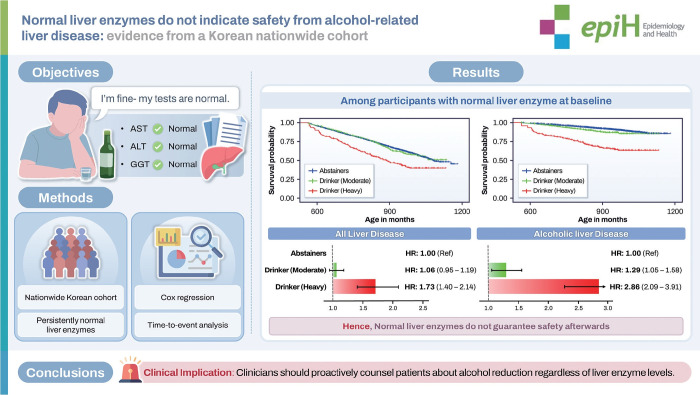


**Table 1.** Characteristics of the study population

Characteristics	Drinking pattern^1^				
	Total	Abstainers	Moderate drinkers	Heavy drinkers	p-value
Total	19,035 (100)	14,479 (76.1)	4,070 (21.4)	486 (2.6)	
Gender					<0.001
Men	7,195 (37.8)	3,440 (23.8)	3,287 (80.8)	468 (96.3)	
Women	11,840 (62.2)	11,039 (76.2)	783 (19.2)	18 (3.7)	
Age (yr)					<0.001
40–49	3,712 (19.5)	2,473 (17.1)	1,157 (28.4)	82 (16.9)	
50–59	8,583 (45.1)	6,265 (43.3)	2,110 (51.8)	208 (42.8)	
60–69	4,097 (21.5)	3,365 (23.2)	622 (15.3)	110 (22.6)	
≥70	2,643 (13.9)	2,376 (16.4)	181 (4.4)	86 (17.7)	
Insurance percentile (%)					<0.001
0–30	4,176 (21.9)	3,404 (23.5)	667 (16.4)	105 (21.6)	
31–70	5,819 (30.6)	4,511 (31.2)	1,140 (28.0)	168 (34.6)	
71–100	9,040 (47.5)	6,564 (45.3)	2,263 (55.6)	213 (43.8)	
BMI (kg/m^2^)					0.028
Underweight (BMI<18.5)	479 (2.5)	392 (2.7)	78 (1.9)	9 (1.9)	
Not obese (18.5≤BMI<25.0)	13,461 (70.7)	10,242 (70.7)	2,885 (70.9)	334 (68.7)	
Obese (BMI≥25.0 kg/m^2^)	5,095 (26.8)	3,845 (26.6)	1,107 (27.2)	143 (29.4)	
Smoking					<0.001
Never-smoker	15,281 (80.3)	13,100 (90.5)	2,016 (49.5)	165 (34.0)	
Former smoker	1,379 (7.2)	465 (3.2)	823 (20.2)	91 (18.7)	
Current smoker	2,375 (12.5)	914 (6.3)	1,231 (30.2)	230 (47.3)	
Physical exercise (times/wk)					<0.001
No physical exercise	9,078 (47.7)	7,636 (52.7)	1,217 (29.9)	225 (46.3)	
1–2	5,359 (28.2)	3,444 (23.8)	1,777 (43.7)	138 (28.4)	
3–4	2,684 (14.1)	1,900 (13.1)	723 (17.8)	61 (12.6)	
5–6	702 (3.7)	528 (3.6)	153 (3.8)	21 (4.3)	
Everyday	1,212 (6.4)	971 (6.7)	200 (4.9)	41 (8.4)	
Family history of liver disease					<0.001
No	18,436 (96.9)	14,063 (97.1)	3,902 (95.9)	471 (96.9)	
Yes	599 (3.1)	416 (2.9)	168 (4.1)	15 (3.1)	
Baseline liver enzyme levels (μ/L)					
Aspartate aminotransferase		22.1±5.2	22.5±5.2	24.1±5.6	<0.001
Alanine aminotransferase		18.5±6.5	20.0±6.8	20.0±6.6	<0.001
Gamma-glutamyl transferase		18.1±7.5	26.6±11.6	33.3±12.7	<0.001

Values are presented as number (%) for categorical variables and mean±standard deviation for continuous variables (baseline liver enzyme levels). BMI, body mass index.

1 Abstainers, drink ≤1 time/mo or do not drink; Moderate drinkers, drink ≤2 times/wk; Heavy drinkers, drink ≥3 times/wk.

**Table 2.** HR of liver disease by drinking frequency among individuals with normal liver enzyme levels

Variables	HR (95% CI)^1^
Drinking pattern^2^	
Abstainers	1.00 (reference)
Moderate drinkers	1.06 (0.94, 1.19)
Heavy drinkers^***^	1.73 (1.40, 2.14)
Gender	
Men	1.00 (reference)
Women	1.04 (0.93, 1.16)
Insurance percentile (%)	
0–30	1.00 (reference)
31–70	1.02 (0.92, 1.13)
71–100	0.92 (0.83, 1.02)
BMI (kg/m^2^)	
Not obese (BMI<25)	1.00 (reference)
Obese (BMI≥25)^***^	1.17 (1.08, 1.28)
Smoking	
Non-smoker	1.00 (reference)
Ex-smoker	1.02 (0.86, 1.21)
Smoker	1.01 (0.88, 1.16)
Physical exercise (times/wk)	
No physical exercise	1.00 (reference)
1–2	0.92 (0.84, 1.02)
≥3	1.01 (0.91, 1.11)
Family history of liver disease	
No	1.00 (reference)
Yes^*^	1.27 (1.04, 1.56)

HR, hazard ratio; CI, confidence interval; BMI, body mass index.

1 The model was adjusted for gender, insurance percentile, BMI, smoking status, physical exercise, and family history of liver disease.

2 Abstainers, drink ≤1 time/mo or do not drink; Moderate drinkers, drink ≤2 times/wk; Heavy drinkers, drink ≥3 times/wk.

* p<0.05,

*** p<0.001.

**Table 3.** Subgroup analysis of the association between alcohol consumption frequency and risk of liver disease according to age group and smoking status

Subgroup	Category	Drinking pattern^1^	HR (95% CI)
Age (yr)^2^	40–49	Abstainers	1.00 (reference)
		Moderate drinkers	1.11 (0.86, 1.42)
		Heavy drinkers^*^	1.81 (1.04, 3.16)
	50–59	Abstainers	1.00 (reference)
		Moderate drinkers	1.07 (0.90, 1.26)
		Heavy drinkers^***^	2.05 (1.49, 2.83)
	60–69	Abstainers	1.00 (reference)
		Moderate drinkers	1.16 (0.90, 1.49)
		Heavy drinkers^*^	1.71 (1.12, 2.61)
	≥70	Abstainers	1.00 (reference)
		Moderate drinkers	1.06 (0.73, 1.56)
		Heavy drinkers	1.22 (0.72, 2.06)
Smoking^3^	Never-smoker	Abstainers	1.00 (reference)
		Moderate drinkers	1.04 (0.91, 1.18)
		Heavy drinkers^**^	1.50 (1.12, 2.01)
	Former smoker	Abstainers	1.00 (reference)
		Moderate drinkers	0.95 (0.72, 1.26)
		Heavy drinkers	1.37 (0.90, 2.09)
	Current smoker	Abstainers	1.00 (reference)
		Moderate drinkers	1.01 (0.82, 1.25)
		Heavy drinkers^**^	1.56 (1.18, 2.05)

HR, hazard ratio; CI, confidence interval.

1 Abstainers, drink ≤1 time/mo or do not drink; Moderate drinkers, drink ≤2 times/wk; Heavy drinkers, drink ≥3 times/wk

2 Adjusted for gender, insurance percentile, body mass index, smoking status, physical exercise, and family history of liver disease.

3 Adjusted for gender, insurance percentile, body mass index, physical exercise, and family history of liver disease.

* p<0.05,

** p<0.01,

*** p<0.001.

**Table 4.** Subgroup analysis of HRs for alcoholic and non-alcoholic liver disease according to alcohol consumption frequency

Liver diseases	Drinking pattern^1^	HR (95% CI)^2^
Alcoholic liver disease	Abstainers	1.00 (reference)
	Moderate drinkers^*^	1.29 (1.05, 1.58)
	Heavy drinkers^***^	2.86 (2.09, 3.91)
Non-alcoholic liver disease	Abstainers	1.00 (reference)
	Moderate drinkers	0.95 (0.83, 1.09)
	Heavy drinkers	1.20 (0.91, 1.58)

HR, hazard ratio; CI, confidence interval.

1 Abstainers, drink ≤1 time/mo or do not drink; Moderate drinkers, drink ≤2 times/wk; Heavy drinkers, drink ≥3 times/wk.

2 All models were adjusted for gender, insurance percentile, body mass index, smoking status, physical exercise, and family history of liver disease.

* p<0.05,

*** p<0.001.

## Supplementary materials

Supplementary Material 1. Schoenfeld proportional hazard test results

Supplementary Material 2. Hazard ratio of drinking patterns for either death or the diagnosis of liver diseases

Supplementary Material 3. Kaplan-Meier curves by drinking pattern when considering death as event.
