# Supplementary material for: Normal liver enzymes do not indicate safety from alcohol-related liver disease: evidence from a Korean nationwide cohort
Source: Epidemiol Health. 2026 Jan 22;48:e2026004. doi: 10.4178/epih.e2026004 (PMC13033442; doi:10.4178/epih.e2026004)
Supplement: Supplementary Material 1. — Schoenfeld proportional hazard test results [file epih-48-e2026004-Supplementary-1.docx]

| Supplementary Material 1. Schoenfeld proportional hazard test results | | | |
| --- | --- | --- | --- |
| Covariates | χ²-value | df | p-value |
| drinking pattern | 6.65 | 2 | 0.0360 |
| Sex | 4.9 | 1 | 0.0268 |
| Insurance percentile | 3.95 | 2 | 0.1385 |
| Body weight | 6.03×10^-5^ | 1 | 0.9938 |
| Smoking | 1.71 | 2 | 0.4259 |
| Physical exercise | 8.58 | 2 | 0.0137 |
| Family history of liver disease | 2.04 | 1 | 0.1533 |
| GLOBAL | 2.79 | 11 | 0.0033 |
