# Supplementary material for: Normal liver enzymes do not indicate safety from alcohol-related liver disease: evidence from a Korean nationwide cohort
Source: Epidemiol Health. 2026 Jan 22;48:e2026004. doi: 10.4178/epih.e2026004 (PMC13033442; doi:10.4178/epih.e2026004)
Supplement: Supplementary Material 2. — Hazard ratio of drinking patterns for either death or the diagnosis of liver diseases [file epih-48-e2026004-Supplementary-2.docx]

| Supplementary Material 2. Hazard ratio of drinking patterns for either death or the diagnosis of liver diseases | | | | |  |  |  |  |
| --- | --- | --- | --- | --- | --- | --- | --- | --- |
| Age | Drinking Pattern | HR | 95% CI | |  |  |  |  |
|  | Abstainers | 1.000 |  |  |  |  |  |  |
| All | Moderate drinkers | 1.000 | 0.904 | 1.108 |  |  |  |  |
|  | Heavy drinkers* | 1.510 | 1.266 | 1.802 |  |  |  |  |
|  | Abstainers | 1.000 |  |  |  |  |  |  |
| 40–49 | Moderate drinkers | 1.186 | 0.934 | 1.506 |  |  |  |  |
|  | Heavy drinkers* | 1.845 | 1.075 | 3.165 |  |  |  |  |
|  | Abstainers | 1.000 |  |  |  |  |  |  |
| 50–59 | Moderate drinkers | 0.984 | 0.840 | 1.152 |  |  |  |  |
|  | Heavy drinkers* | 1.846 | 1.366 | 2.495 |  |  |  |  |
|  | Abstainers | 1.000 |  |  |  |  |  |  |
| 60–69 | Moderate drinkers | 1.089 | 0.879 | 1.349 |  |  |  |  |
|  | Heavy drinkers* | 1.463 | 1.012 | 2.114 |  |  |  |  |
|  | Abstainers | 1.000 |  |  |  |  |  |  |
| 70≤ | Moderate drinkers | 1.032 | 0.803 | 1.337 |  |  |  |  |
|  | Heavy drinkers | 1.198 | 0.872 | 1.646 |  |  |  |  |
| * statistically significant associations; HR, hazard ratio | | | |  |  |  |  |  |
| Abstainers, drink ≤ 1 time/month or do not drink; Moderate drinkers, drink ≤ 2 times/week; Heavy drinkers, drink ≥ 3 times/week | | | | | | | | |
| All models were adjusted for sex, insurance percentile, body weight, smoking status, physical exercise, and family history of liver disease. | | | | | | | | |
