# Supplementary figures and images for: Normal liver enzymes do not indicate safety from alcohol-related liver disease: evidence from a Korean nationwide cohort
Source: Epidemiol Health. 2026 Jan 22;48:e2026004. doi: 10.4178/epih.e2026004 (PMC13033442; doi:10.4178/epih.e2026004)

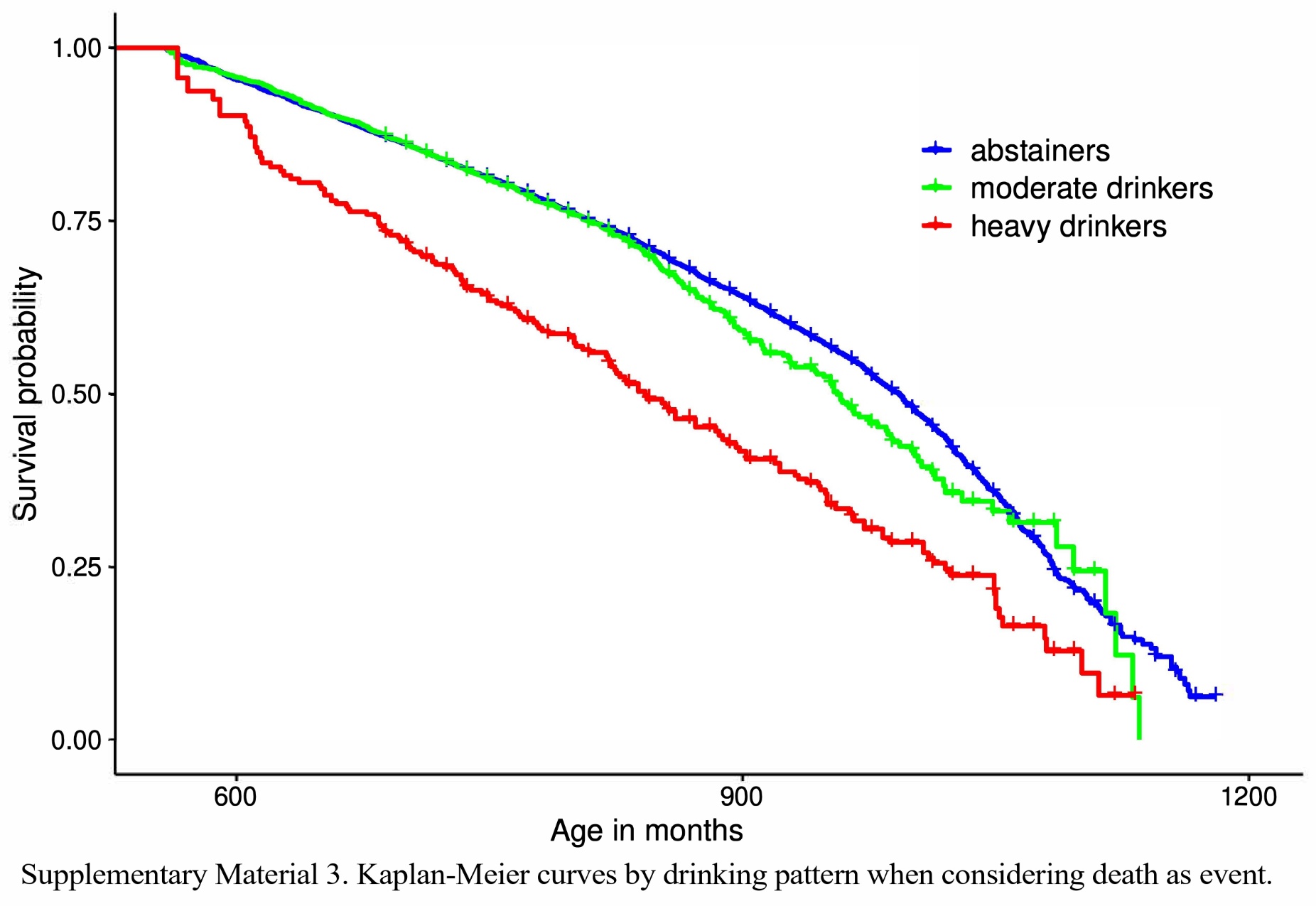

Supplement: Supplementary Material 3. — Kaplan-Meier curves by drinking pattern when considering death as event. [file epih-48-e2026004-Supplementary-3.docx]
